# Early clinical indices predicting functional survival in severely head-injured patients

**DOI:** 10.1186/cc9736

**Published:** 2011-03-11

**Authors:** M Zouka, G Tsaousi, E Anastasiou, E Geka, E Euthymiou, I Soultati, M Giannakou

**Affiliations:** 1 AHEPA University Hospital, Thessaloniki, Greece

## Introduction

Given the burden of disability arising from severe traumatic brain injury (TBI) [1], plain assessment of mortality certainly underestimates the impact of TBI. Therefore, risk prediction models need to provide poor neurological outcome estimates other than mortality. The aim of the study was to determine whether a simple combination of early clinical indices may be predictive of disability after ICU discharge.

## Methods

A prospective study enrolling 133 patients (109 male/76 female) with TBI (associated or not with multiple trauma) and GCS ≤8 admitted to our ICU. Demographics, acute care preadmission factors (hypotension and hypoxemia), injury severity (GCS, ISS, RTS, pupil reactivity, CT scan grade) and acute physiological disturbance (APACHE II - 24 hours, SOFA) were evaluated. According to functional outcome (GOS) upon ICU discharge, two subgroups of patients were identified: GOS 4 to 5 (favorable outcome), and GOS 1 to 3 (poor outcome). Independent *t *test, Mann-Whitney test, logistic regression, ROC curve and chi-squared analyses were used for statistical purposes.

## Results

Data are presented in Table 1. Overall mortality was 32.3% (*n *= 43). Logistic regression analysis identified APACHE II (*P *= 0.004), CT scan grade (*P *= 0.002) and pupil reactivity upon ICU admission (*P *= 0.01) as the strongest predictors of functional outcome. Area under the ROC curve for APACHE II score was 0.841 (95% CI: 0.767 to 0.899, *P *< 0.0001).

**Table 1:** 

Parameter	GOS 1 to 3 (*n *= 56)	GOS 4 to 5 (*n *= 77)	*P *value
Age (years)*	42.9 ± 22.8	31.9 ± 14.8	0.002
Hypotension (%)	28.6	9	0.03
Hypoxia (%)	23.2	5.2	0.01
ICU pupils (abnormal) (%)	35.7	1.3	0.000
CT scan grade >2 (%)	64.3	37.6	0.000
ISS*	35.9 ± 14.7	23.9 ± 10.3	0.000
APACHE II*	222.2 ± 5.5	15.03 ± 5.3	0.000
GCS*	4.9 ± 1.8	6.5 ± 1.8	0.000
RTS*	4.1 ± 1.3	5.02 ± 1.3	0.04
SOFA*	6.5 ± 3.0	4.1 ± 2.1	0.005

*Data presented as mean ± SD.

## Conclusions

Acute physiological disturbance, poor preadmission clinical data and neurological signs, presence of severe intracerebral injuries combined with additional extracerebral injuries and advanced age, seem to be powerful determinants that adversely influence the early course of recovery and functional survival of patients with sustained severe TBI. Among them APACHE II, CT scan grade and pupil reactivity upon ICU admission were identified as the strongest early prognostic indicators.
